# Supplementary material for: Rapid assessment of hand reaching using virtual reality and application in cerebellar stroke
Source: PLoS One. 2022 Sep 29;17(9):e0275220. doi: 10.1371/journal.pone.0275220 (PMC9522266; doi:10.1371/journal.pone.0275220)
Supplement: Attachment — Submitted filename: Response to Reviewers 2.docx [file pone.0275220.s007.docx]

Thank you for the additional attentive comments on our manuscript. We have addressed the comments below:

Reviewer #2:

1. *The authors have addressed all my comments and I believe that the manuscript now reads more focused and clearer. I still have some remaining comments, some of which arise due to the revisions whereas a few others concern issues that I could have spotted in the first round. Apologies for not bringing these up earlier. All lines mentioned below concern the track-changes document.*
   a. Thank you again for the valuable comments, we agree that the paper is more streamlined and easier to follow.
2. *Abstract: the first lines of the abstract greatly focus on the ‘perception’ side and the limitations of the traditional experiments on perception. Though this true, this study here focuses more on action/behavior. I would encourage the authors to either bring the ‘action’ part more explicitly, or even discuss the perception-action part altogether (than only talking about ‘perception’).*
   a. We have expanded the beginning of the abstract to emphasize the relevance of both perception and action to this manuscript.
3. *Introduction, paragraph 1 (lines 55-68): Similarly here, the focus appears to be on ‘perception’. Yes, perception research started with rather passive, highly controlled designs, but this has now evolved to more naturalistic experiments. In the same line, ‘action’ research started with constrained paradigms, such as the studies of Jeannerod on grasping, but has evolved to whole-body tracking in the nature, as the authors also cite the work of Matthis and colleagues. Therefore, as in the abstract, I recommend also here to focus also on action, not only on perception.*
   a. The introduction now contains additional mention of the interplay of action and perception as well as past limitations of action research, such as small or restricted movements.
4. *Methods, lines 149-154: Please introduce the participants of experiment 3 somewhere here. This is important otherwise the term ‘healthy’ in line 152 reads odd, while doing so will facilitate reading of the subsequent section (e.g., lines 161-162).*
   a. An introduction to the participants in Experiment 3 has been added at the beginning of the Methods section.
5. *Line 207: It remains unclear whether the error is measured only on the lateral direction or the vertical direction is also accounted. Please make this more explicit.*
   a. Further clarification has been added that the error accounts for error in both the horizontal and vertical direction.
6. *Line 209: did the reaching time indeed consider the moment when the participant ‘touched the target’? In other instances (e.g., lines 199-201), it is mentioned that the trial ended when the participant crossed the arc, implying that this would be used as the reaching time. If reaching time is indeed measured based on when the participant touched the target, what happened in trials in which the participant did not touch the target? If the reaching time is measured based on the time when the participant crossed the arc, this line needs to be revised (as also other instances throughout the manuscript, for instance line 232, 262, and possibly elsewhere).*
   a. Thank you for pointing out this discrepancy, we have clarified throughout the paper that the reaching time begins when the target appears and end when the index finger passes through the arc, rather than when it specifically touches the target.
7. *Lines 226-227: Could you please clarify whether the 100 trials of one of the conditions were presented before/after the 100 trials of the other condition, or whether the two conditions were randomly presented randomly interleaved across the 200 trials?*
   a. We have clarified that the two conditions of trials are randomly interspersed together in all experiments.
8. *Line 276: “…of their accuracy”. I think the authors here should state “…of their endpoints”. Also, is precision calculated as the SD along the lateral direction only? Please clarify in the manuscript.*
   a. We have clarified here that endpoint accuracy is the metric being discussed.
9. *Lines 309-312: These here read as ‘methods’ but are presented in the ‘results’. My recommendation is to define the three measures (accuracy, precision, reaching time) in the Methods, one after the other, so that it easier for the reader to follow the analysis.*
   a. We have reorganized this information so the beginning of the Statistical Analysis section now defines the three metrics of reaching accuracy, reaching precision, and reaching time together.
10. *Line 326: “of this measure”. Which measure? The previous part refers to two different variables, accuracy and precision. Why not calculate precision and reaching time also on the basis of the first 25 trials? Then the authors, for each experiment, would have a first paragraph with results about the three measures (accuracy, precision, time) considering all trials, and a second paragraph with the respective results when considering only the first 25 trials. Then the results would be easier to follow and interpret.
    If the authors would like to focus mainly on accuracy, this should become more explicit, ideally with a reason why.*
    a. Thank you for pointing out this unclear phrasing; we have clarified that we referred to the sensitivity of the experiment to assess reaching accuracy. We have also stated that reaching accuracy was the primary outcome measure because it has the greatest clinical significance and effect on quality of life and independence.
11. *Lines 333-335: Does this refer to reaching time considering all trials or only the first 25 trials of each condition?*
    a. This line has been moved to clearly indicate that it is referring to reaching time across all 100 trials.
